# A novel methodology to study the release of fragmented fibres, including microplastics, in laboratory washing conditions

**DOI:** 10.1038/s41598-026-41563-7

**Published:** 2026-03-01

**Authors:** Ángel Palacios-Marín, Alma Victoria Palacios-Marín, Muhammad Tausif, Wernher Brevis

**Affiliations:** 1 Department of Hydraulic and Environmental Engineering, Escuela de Ingeniería, Pontificia Universidad Católica de Chile, Santiago, Chile; 2 School of Design, University of Leeds, Leeds, UK

**Keywords:** Fragmented Fibres, Textile fibres, Microplastics, Particle Image Velocimetry, Reynolds number, Washing machine, Engineering, Materials science

## Abstract

Fragmented fibres, including fibrous microplastics, are increasingly recognised as a major pollutant originating from textile laundering. While many studies have quantified fibre release, the mechanisms underlying fibre fragmentation and detachment remain poorly understood, partly due to the lack of techniques to capture fibre–flow interactions under controlled conditions. This study introduces a novel methodology for the simultaneous characterisation of turbulent flow and textile fibre dynamics under simulated washing conditions. The approach combines a Von Kármán Swirling Flow apparatus, which generates reproducible shear and turbulence, with dual high-speed imaging to record both the fluid, analysed using Particle Image Velocimetry, and the fibres, analysed using optical flow. Proof-of-concept experiments with polyester and cotton yarns demonstrate that the method can capture fibre motion, deformation, and protrusion in synchrony with surrounding flow fields. A Reynolds number definition for top- and front-loading washing machines is also proposed, providing a basis for comparing washing conditions. This reproducible, non-invasive framework establishes a foundation for systematic investigations of fibre behaviour during laundering by providing mechanistic insight into fibre breakage and release processes that underpin textile-derived pollution, which is not covered by existing gravimetric methods that quantify mass release from textiles.

**Supplementary Information:**

The online version contains supplementary material available at 10.1038/s41598-026-41563-7.

## Introduction

Textile fibres from synthetic, regenerated, and natural sources have been detected in virtually every ecosystem across the globe^1,2^. Their pervasive presence has triggered significant concern due to their potential environmental hazards, which have been actively studied across various disciplines, as they impact aquatic ecosystems, threaten biota, and serve as hosts and carriers for pollutants and toxic compounds^2,3^. Research has shown a definitive link between these fibre pollutants on shorelines and the laundering of clothing, identifying a critical and previously underestimated source of environmental microplastic (MP) pollution^1^. Textile abrasion during laundering is now estimated to account for 35% of the primary microplastics released into the ocean, underscoring the substantial impact of textile-derived marine pollution^4^. MPs originating from textiles are often associated with synthetic fibres. However, all textiles, whether synthetic, regenerated or natural, release fragmented fibres into the environment^5^. This broader category, referred to as Fragmented Fibres (FF), encompasses fibres released from any textile source, providing a comprehensive framework for understanding fragmented fibre pollution across all material types, including plastics^5^.

The global production of textile fibres has doubled between 2000 and 2020, and is estimated to increase by a further 30% by 2030^6^. The rising increase in textile consumption amplified the prevalence of FF, making them a pollution problem and a potential hazard impacting the health of living organisms. Hence, it is imperative to understand the root causes and develop effective mitigation strategies^6^.

Several authors have studied fibre shedding from textile washing using accelerated laundering testing instruments and domestic washing machines^1,3,7–9^. The absence of standardised nomenclature and testing methods in studies on FF released from textiles during washing has resulted in a lack of consistency, making it challenging to compare results across different research studies directly^5,10^.

Recent efforts to address the ambiguity surrounding fibre fragment pollution have led to the adoption of more precise nomenclature and the establishment of international standards for the generation and collection of FF from textile washing. The term “fibre fragments” has been adopted by international test methods^11,12^ to specifically refer to pollutants shed from textiles, separating them from “microfibres”, which describe fibres manufactured industrially at a micro size^11^. There are two standard methods (AATCC TM212-2021 and BS EN ISO 4484-1:2023) for measuring material loss from textiles, outlining a systematic procedure for washing textile samples using accelerated laundering instruments to generate, filter and collect released material, followed by mass-based quantification of the collected material^11,12^. Additionally, BS EN ISO 4484-3:2023 describes procedures for washing and collecting textile particles from domestic laundering machines.

While establishing international testing standards is a significant step in addressing many key needs, both standards are descriptive quantification methods that do not explain the underlying causes or mechanisms behind fibre shedding. These testing standards measure the increase in filter mass, which represents total mass gained on the filter and may include fibres, fibre fragments, and other particulate matter. The identification, counting, and dimensional classification of released components require complementary analytical procedures, as described in BS EN ISO 4484-2^13^. While the standards provide essential quantitative benchmarks, the gravimetric approach prevents elucidation of the underlying mechanisms driving fibre fragmentation and release. As a result, the standards offer limited insights for optimising textile structures or washing conditions to mitigate fibre release at source. Consequently, the paucity of mechanistic research into the root causes of fibre fragmentation and release highlights the need for novel approaches to a fundamental understanding of fibre damage. The current work presents an experimental method to better understand the fundamental factors and mechanisms driving fibre breakage and detachment during laundering, aiming to improve the design of textiles and washing machines, thereby reducing the detachment of fragmented fibres.

Most of the existing literature on FF shedding from textiles during washing is focused on the impact of textile materials and structures^14–19^ or the influence of laundering parameters on the release of FF^20–25^. However, there is a paucity of work on the understanding of the physical effects of laundering on fibre breakage and the mechanical action imparted on textile fibres during washing^26^. The existing studies do not provide in-situ, simultaneous, time-resolved measurements of fibre deformation and motion under realistic turbulent forcing, limiting the ability to link observed fibre release to underlying physical stresses.

Zambrano et al. proposed that a fabric’s shedding capacity is influenced by its propensity to form fuzz, describing fuzz formation as a process involving a fibre with a loose end extending out of the fabric^6,14^. While some FF may originate through this mechanism, pilling alone may not fully explain the phenomenon. Despite the resistance to fuzz formation^5,27^ of continuous filament fabrics, they have been reported to release FF by various authors^9,15,24,28,29^. Existing testing methods are limited to quantification and do not provide the physical evidence necessary to validate the proposed mechanisms. From a physical perspective, the interaction between flow, fibre motion and fibre breakage may be central to understanding the release of FF. Therefore, simultaneous observation of flow dynamics and fibre response is essential to fully elucidate these mechanisms.

The present work introduces a novel methodology designed to simultaneously investigate the physics of the flow properties and fibre dynamics as a coupled system, providing a comprehensive understanding of fibre breakage (generation) and detachment (release) mechanisms. This novel two-dimensional approach studies fluorescent yarns illuminated by a laser sheet and visualised with high-speed cameras for further analysis with an image velocimetry system. The proposed facility generates a controlled Von Kármán Swirling Flow (VKSF), allowing a precise observation of fibre-flow interaction under dynamic conditions. Particle Image Velocimetry (PIV), a non-invasive method that computes velocity vectors between consecutive snapshots of tracer particles seeded in the flow^30^, is employed to provide detailed insights into the flow characteristics around the fibres. In contrast, fibre motion is tracked using optical flow, an extensively used computer vision technique. To the author’s knowledge, the proposed methodology enables, for the first time, direct visualisation and analysis of fibre motion from a yarn under simulated washing conditions. The robust, innovative approach allows the study of synthetic, regenerated, and natural fibres under various flow conditions across different textile structures and exposure environments.

## Materials and methods

### Von kármán swirling flow apparatus

**Fig. 1:**
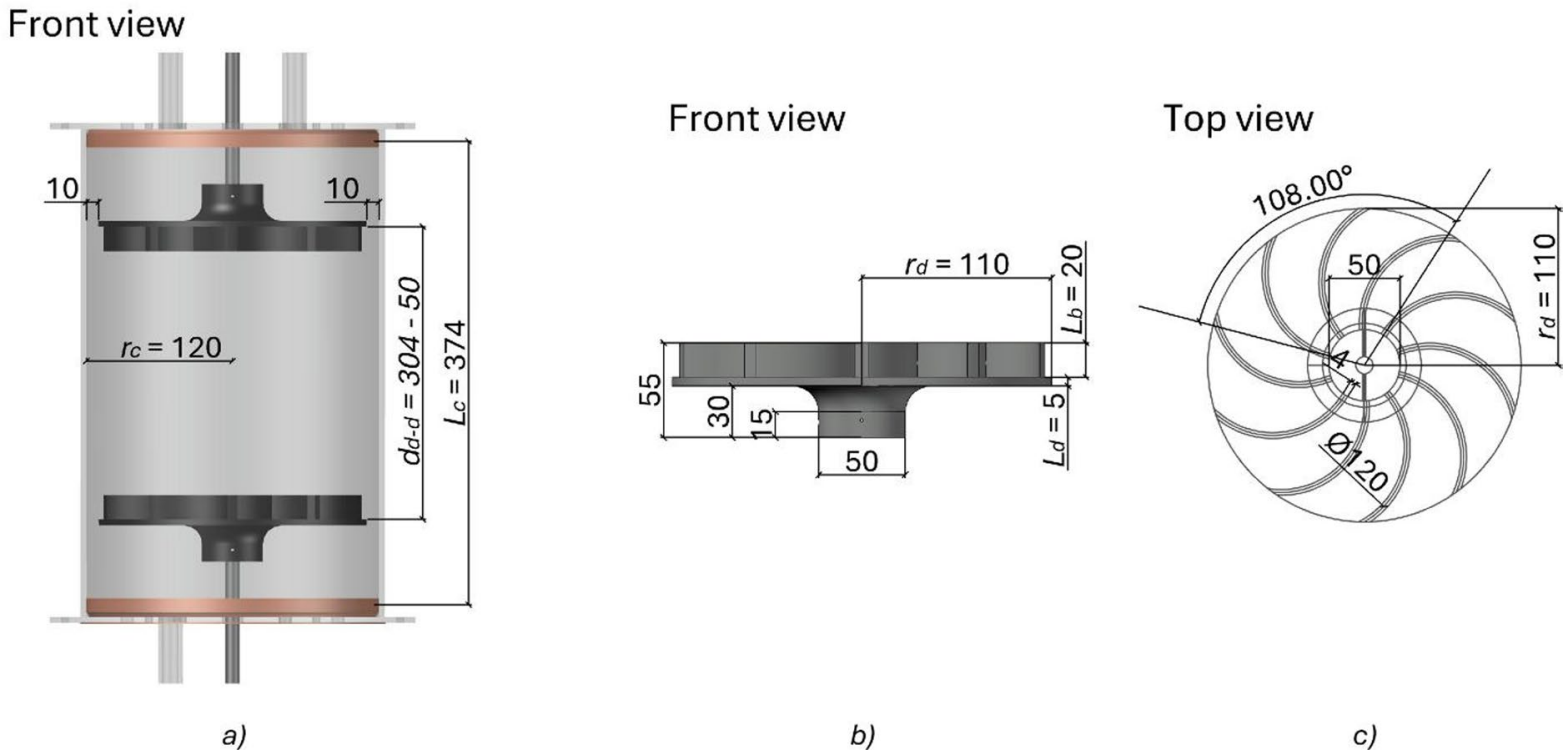
VKSF apparatus formed by an acrylic cylinder containing two counter-rotating impellers (black) at a). The salmon colour represents pistol seals. Impellers front and top view in the b) and c), respectively. The impellers are manufactured in aluminium and anodised in dark blue to avoid light reflection. All dimensions are in mm, except angle measurements displayed in degrees.

Experiments are carried out in a Von Kármán Swirling Flow generated between two counter-rotating impellers in an acrylic prefabricated cylinder, allowing reproducible flow conditions. The flow generated between the impellers consists of two counter-rotating toroidal vortices that create a high-shear layer in the cylinder’s centre. The cylinder has an interior radius $\:{r}_{c}=120$ mm and a usable height $\:{L}_{c}=374$ mm, giving a volume $\:{V}_{c}=16.92$ litres. The impellers were manufactured in aluminium using a computer numerical control (CNC) machine to ensure symmetry and anodised in dark blue to avoid light reflection. Each impeller is formed by a bottom disk of height $\:{L}_{d}=5$ mm and a radius $\:{r}_{d}=110$ mm, with 10 blades with a curvature radius of $\:60$ mm rotated $\:30^\circ\:$ respect to the disc rim, and a blade height $\:{L}_{b}=20$ mm, ensuring that the boundary layer on the impellers is smaller than the blade’s height. Therefore, the turbulence is generated by inertial forcing, and the energy dissipation rate $\:\epsilon$ is independent of the fluid viscosity^31^. The distance between the inner disk faces ($\:{d}_{d-d})$ can vary from 50 mm to 304 mm, for a ratio of $\:4.4>2{r}_{d}/{d}_{d-d}>0.72$. Each impeller is driven by an overhead stirrer OHS 60 Advance (VELP Scientifica), delivering an angular velocity $\:{\Omega\:}/2{\uppi\:}$ between $\:0.5$ and $\:33.3$ Hz. Torque and angular velocity are monitored and adjusted in real-time by SpeedServo™ technology, ensuring constant velocity. A controller program^32^, available for free download, was written to communicate with the overhead stirrers, monitoring and recording velocity, torque, and fluid temperature in real-time at one-second intervals. The VKSF apparatus is enclosed in an acrylic octagon filled with the same fluid as the cylinder to avoid image distortion^33^ for velocimetry system recordings. The cylinder is bounded by two acrylic lids with pistol seals for the cylinder walls and rotating shaft seals for the impeller shafts, ensuring no contamination is introduced to the fluid. An illustration of the VKSF apparatus and the impellers is shown in Fig. 1.

Materials.

Given the depth of field of the cameras (the distance range that appears sharp in an image) used for experimentation, the detachment of FF was studied by directly visualising the fibres from a single yarn. Yarns are bundles composed of natural and/or extruded staple fibres and/or filaments^34^. They constitute the fundamental structural element of any geometrically organised fabric. The yarns are formed by twisting parallel fibrous strands, which enhances the lateral cohesion of the fibrous assembly^34^. Two types of fibres were selected to evaluate the feasibility of the proposed method: one synthetic, polyester (PET), and one natural, cotton (CO). These fibres were chosen because they are the most widely used in apparel globally, with PET accounting for 57% of total fibre production and cotton accounting for 20% of global fibre output^35^. They were converted into yarns by ring spinning, which remains the predominant and most versatile technology for producing staple yarns^36^. The yarns were custom-manufactured in the laboratory for this study, with detailed specifications provided in Table 1.

**Table 1:** Textile details of experimental yarns.

Fibre ID	Fibre composition	Fibre thickness	Linear density (Ne)	Spinning method	Twist Multiplier (TM)
PET	100% PET	1.2 denier	30/1	Ring-spinning	3.75
CO	100% Cotton	4.5 micronaire			

#### Yarn preparation and dyeing procedures

To achieve the fluorescence required for effective laser visualisation, both yarn types underwent a preparation process that included scouring and dyeing. A thread spool was used for each sample to hold 100 m of yarn. The plastic spools were modified with holes throughout their structure to facilitate the flow of the soaping and dyeing baths into the yarn. The yarn was loosely wound on the spool to ensure fluid permeability while preventing free movement and tangling. The thread spools with samples were conditioned overnight by placing them in standard atmospheres with a relative humidity of 65 ± 2% and a temperature of 20 ± 2 °C, as stipulated in BS EN ISO 139:2005^37^. After conditioning, the average weight of each sample was measured using a Mettler Toledo AE160 analytical balance (Mettler Toledo Inc.), with an accuracy of five decimal places (0.00001 g). The sample mass was used to calculate the required quantities of reagents for the scouring and dyeing processes. Both procedures were executed using a Pyrotec3 2000 laboratory dyeing machine (Roaches International).

Yarns were scoured as a preparation for dyeing to remove impurities and residual treatments from the manufacturing process. A soaping solution of deionised (DI) water and a non-phosphate standard powder detergent without optical brightener or enzymes (48349E: AATCC 1993 Standard Detergent; AATCC) was prepared at a liquor ratio of 15:1 relative to fibre mass (RFM), and a detergent concentration of 2 g/L. Thread spools were placed in Pyrotec canisters containing the solution, heated to 100 °C at 5 °C/min, held for 20 min, then cooled to 40 °C at 3 °C/min, and rinsed thoroughly.

Sudan II (Solvent Orange 7) was selected for PET dyeing due to its strong solid-state fluorescence, attributed to its aggregation-induced emission properties. It exhibits a broad excitation range (250–600 nm), with peak responses in the 500–600 nm region^38^. Although limited information is available concerning the emission characteristics of Sudan II when applied to textile substrates, its proven compatibility with plastic materials and the alignment of its excitation range with the laser parameters used in this study support its selection. A preliminary test was conducted, and the results confirmed the suitability of the dye on PET fibres for the experiments. The dyeing solution was prepared using DI water at a 15:1 liquor ratio RFM, containing 2% dispersing agent (lignosulfonic acid sodium salt; Sigma-Aldrich Solutions) and 1.5% dye (Sudan II; Fisher Scientific UK Ltd), both calculated RFM. The dispersing agent was stirred into water until fully dispersed. The dye was dissolved in acetone and gradually added to the solution under continuous stirring. Pyrotec canisters with the solution and thread spools were heated at 2 °C/min to 130 °C, maintained for 60 min, then cooled to 40 °C at 3 °C/min. Excess dye was removed via a reduction-clear process using 1 g/L sodium dithionite and 1 g/L sodium hydroxide in DI water (15:1 liquor ratio, RFM), heated to 70 °C at 2 °C/min for 30 min, then cooled to 40 °C at 3 °C/min. Spools were then rinsed thoroughly and dried overnight.

Rhodamine B is a water-soluble fluorescent xanthene dye utilised for staining fluorescent applications^39^. Sulfonated derivatives of Rhodamine B find utility as dyes for wool and polyamide materials^40^. This characteristic prompted its selection for application in cotton, displaying an excitation at 525 nm and emission at 548 nm^41^, aligning with the requirements of the laser beam and optical filter used in the experiment. The dyeing bath was prepared with DI water at a 15:1 RFM liquor ratio, containing 1.5% dye (Rhodamine B; Fisher Scientific UK Ltd) and 80 g/L sodium chloride (NaCl; Fisher Scientific UK Ltd). Thread spools in dyeing canisters with the solution were heated to 100 °C at 2 °C/min for 60 min, then cooled to 40 °C at 3 °C/min and rinsed. A reactive soaping treatment followed, using DI water at a 15:1 liquor ratio (RFM) with 1% anionic emulsifier (Hostapal BV, conc. Ultrus LLC), heated to 100 °C at 2 °C/min for 30 min, then cooled to 40 °C at 3 °C/min. Samples were rinsed thoroughly and air-dried overnight.

### Yarn mounting

**Fig. 2:**
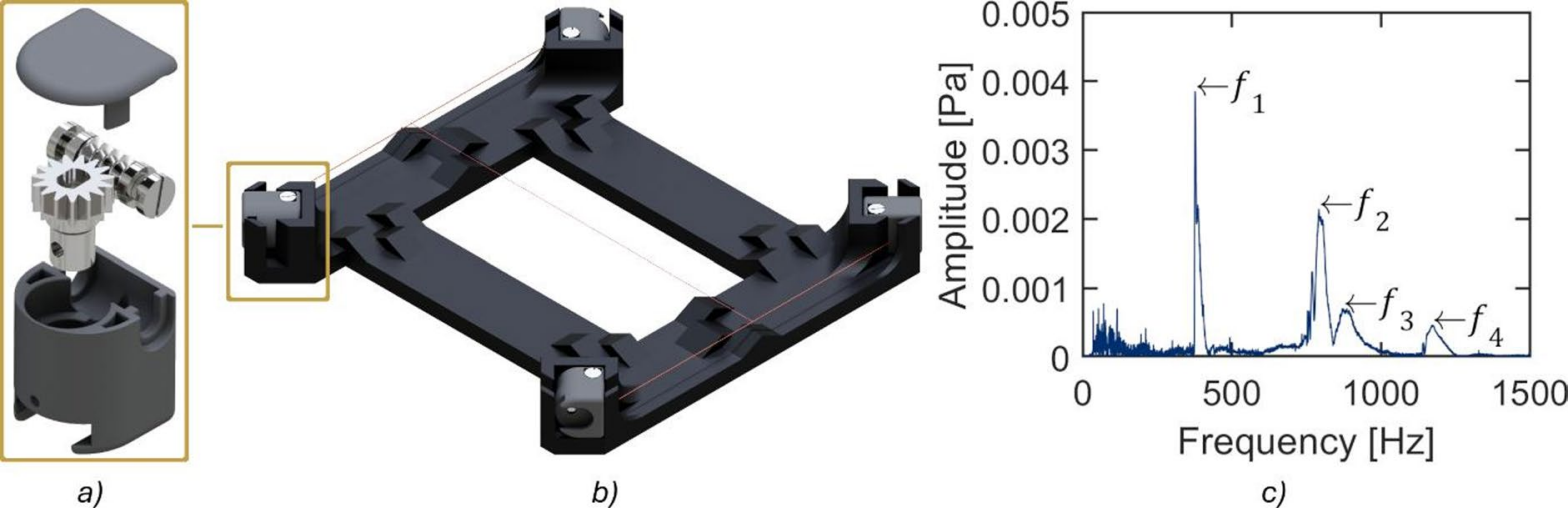
Complete 3D-printed mounting system in b) with a yarn tied to two transverse copper wires. Detailed 3D-printed tension holder (one of four) to adjust the tension of copper wires in a). The worm drive, shown in grey, is fabricated from steel with an orifice to hold copper wires. The FFT obtained from audio recordings used to adjust the tension of copper wires is displayed in c). The first four frequencies (in Hz) are 𝑓_1_ = 377, 𝑓_2_ = 791, 𝑓_3_ = 873, 𝑓_4_ = 1172.

Fluorescent-dyed yarns are positioned in the centre of the acrylic cylinder using 3D-printed resin holders and copper wires. The holders, shown in Fig. 2a), encage a metal worm drive with a dowel pin fixed to the worm wheel. The dowel pin has a hole in its end to hold a 0.28 mm copper wire. The pin hole coincides with orifices in both sides of the 3D-printed holders, allowing the copper wire to pass through. To tension, each end of the copper wire is gripped by two holders. Two pairs of holders with copper wires between them are sited in a second 3D-printed positioner, shown in Fig. 2b), before mounting the yarn.

The frequency method^42^ is used to determine the tension of copper wire. This method establishes the relationship between cable tension and the frequency of cable vibration produced when an instantaneous force is applied, causing the cable to generate a vibration wave that propagates into the surrounding air (phonates). Cable physical properties and vibration frequency are substituted in Eq. (1)^42^, determining its tension. In this study, $\:T$ is wire tension, $\:m$ is mass per unit length, $\:l$ is wire length, $\:E$ is Young modulus, $\:I$ is area moment of inertia. Audio recordings of the wire vibrating are used to determine the frequency $\:{f}_{n}$ and the peak frequency number $\:n$ after a Fast Fourier transform (FFT). An example of a frequency spectrum of an FFT can be seen in Fig. 2c) with several peak frequencies and their corresponding $\:n$th numbers.

1 $$\:T=4{\pi\:}^{2}m{l}^{2}\frac{{f}_{n}^{2}}{{\left(n\pi\:\right)}^{2}}-\frac{EI}{{l}^{2}}{\left(n\pi\:\right)}^{2}{f}_{n}$$

A previous research^42^ has shown the feasibility of using a mobile phone microphone instead of a professional microphone for the frequency method. The authors demonstrated that the first peak frequencies recorded with a mobile phone can be used to determine cable tension accurately. Therefore, this study relied on its use. Finally, to adjust the tension in both wires, a desired $\:T$ was set and its corresponding $\:{f}_{n}$ and $\:n$ were reached by turning the worms, considering that the rest of the variables in Eq. (1) remain unaltered since the temperature in the laboratory is relatively stable, and the same kind of copper wire was used in all the experiments.

After adjusting wire tensions, a dyed yarn is tied with a 180 g weight to ensure repeatability. First, one end of the yarn is tied to the centre of one of the copper wires. Then, in the second yarn end, a $\:180$ g weight is temporarily fixed to tension before tying the second end to the other copper wire. An example of the 3D-printed mounting system with copper wires and yarn is displayed in Fig. 2b).

Afterwards, two laser light sheets, pointing to the cylinder’s centre in the horizontal and vertical directions, were used to set the yarn position. In each experiment, the mounting system, along with copper wires and yarn, is settled until the yarn is entirely illuminated by both laser lights, with wavelengths of 650 nm (red light) and 532 nm (green light). The large 3D-printed positioner is withdrawn, and the four holders containing the worm drives remain in place via suction pads previously inserted into the holders’ front ends. Suction pads are not shown in Fig. 2c) to allow a clearer design display.

### Flow and fibre simultaneous visualisation and analysis

Flow and fibre are visualised with an image velocimetry system located at the Department of Hydraulic and Environmental Engineering at the Pontifical Catholic University of Chile. The system included a 5 W 532 nm laser, laser optics, two high-speed 12-megapixel TSI PowerView model 630,097 cameras (4096 × 3072 pixels in the horizontal and vertical direction, respectively), able to capture up to 181 fps at full definition, cylinder lenses, 50 mm camera lenses, Manfrotto centre ball heads, and a 545 nm optical filter. Laser and cameras were controlled and synchronised by a TSI synchroniser model 610,036 with a precision of 2 ns. Synchroniser and overhead stirrers were connected to a computer to program, start, and record the experiments. Each camera, which can increase the frame rate by reducing image height, is connected to the same computer via a 4-channel Komodo CXP frame grabber over PCI Express. The computer had 196 GB of RAM, of which 170 GB were available for image capturing.

**Fig. 3:**
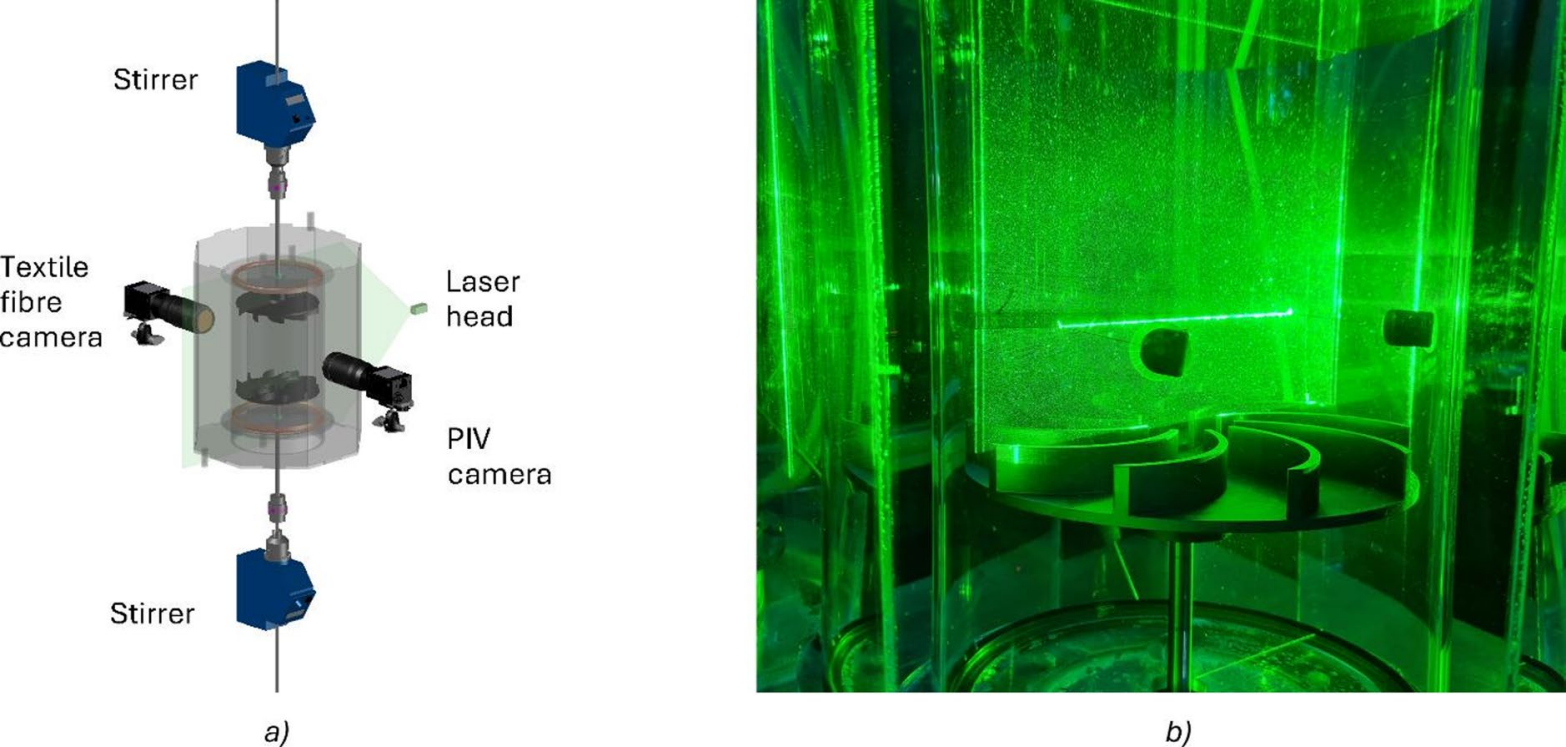
Simultaneous flow visualisation. The cameras and laser arrangement are illustrated in a). Each camera is positioned to face one side of the cylinder, with a 180 ° angle between them. The laser is placed at 90 ° with respect to either camera. A photograph of the yarn (the straight, bright green line) and tracer particles is shown in b). The 3D-printed tension holders, visible in black in b), remain in place during the experiment.

Two image series were recorded simultaneously from each experiment: one for the flow and one for the fibre. Each camera is positioned 180 ° from each other, pointing to the cylinder centre where the shear layer and yarn are located. Illumination is achieved by a light sheet generated by the laser, oriented at 90 ° to the cameras. The laser operated in continuous mode, controlling image acquisition by adjusting the diaphragm exposure time of the cameras. An illustration of the cameras and laser placement is displayed in Fig. 3a). A photograph with tracer particles and a yarn illuminated by the laser is shown in Fig. 3b). The recording plane measurements and camera exposure time are displayed in Table 2. The resolution in all the experiments is 0.031 mm/pixel, and the recording time was 41 s.

**Table 2:** Acquisition frequency and recording plane measurements.

Overhead stirrer motor frequency [Hz]	Acquisition frequency [Hz]	Diaphragm exposition [µs]	Resolution [pixel]	Plane size [mm]
0.500	400	450	4096 × 1376	126.4 × 43.1
0.667	512	410	4096 × 1056	126.4 × 33.1
0.833	625	200	4096 × 864	126.4 × 27.1

Flow velocity is determined using PIV, a widely known non-invasive method that computes velocity vectors for consecutive snapshots. The process consists of capturing an image series of tracer particles seeded in the flow, in this case, 8 to 12 μm hollow-glass spheres fabricated by TSI, illuminated by a light source (laser). Tracer particle density is 1.1 ± 0.05 g/cm^3^. Particle seeding was carefully monitored to ensure 5 to 7 particles per final interrogation window and a particle displacement of no more than 25% of the interrogation window size for optimal results. Also, any particle was displayed for at least 3 pixels to allow subpixel accuracy. Image pre-processing included subtracting the mean image of each series, applying contrast-limited adaptive histogram equalisation with a window of 64 pixels, a denoising Wiener filter with a size of 3 pixels, and an intensity capping filter^43^, all of which were necessary due to yarn lightness. After pre-processing, each image is divided into interrogation windows to compute cross-correlations of seeded particles’ brightness between sequential images, allowing window deformation for better results^44^, obtaining velocity vectors from the most probable displacement (i.e., the higher cross-correlation). This latter process can be repeated with sub-windows, with the initial displacement used as a preliminary guess before calculating the next cross-correlation. Processing was performed using PIVlab^45^, a free MATLAB toolbox that has yielded robust results^46^. The configuration was 4 passes with a 50% step size. Pass 1 had an initial interrogation window of 128 pixels. Passes 2 and 3 had 50% of the previous step size, i.e., 64 and 32 pixels, respectively. Pass 4 was a repetition of pass 3, yielding the smallest interrogation window size of 32 × 32 pixels with a vector size of 16 × 16 pixels. Further information on the PIV method can be found in specialised bibliography^30^.

After obtaining the velocity vectors, post-processing was performed. For velocity vectors, missing fields are interpolated, then a local median filter^47^ is applied with a threshold of 3 times the mean surrounding vectors, and a velocity smoothing^48^ is done. Image distortion caused by the camera lens and unit conversion from pixels/frame to m/s were corrected using an in-house-written algorithm with biharmonic interpolation. Calibration points used in the interpolation were obtained from a plaque covering all the recording area with a separation between points of 5 mm. Here again, each point covered at least 3 pixels to get sub-pixel accuracy. Using biharmonic functions gave a maximum error of less than 10^− 12^ mm in both horizontal (𝑥) and vertical (𝑦) directions, including the further points where the distortion is greater (image edges). A point correction with a 5th-degree polynomial function was compared to biharmonic functions for testing purposes. Results showed an error of up to 0.09 mm in the 𝑥 direction and 0.06 mm in the 𝑦 direction using the polynomial case. Biharmonic interpolation was preferred, given the size of the particles and the yarn in the image, and considering that the recording plane measures only 126.4 mm in the longest direction, even though computing time from polynomial interpolation is a small fraction with respect to biharmonic interpolation.

Yarns were recorded using wavelength differentiation. One of the high-speed cameras, equipped with a 545 nm filter, recorded PET and CO fluorescent dyed yarns. Cotton was dyed with Rhodamine B, with excitation at 525 nm and emission at 548 nm. Sudan II dye was used for PET fibres, which has a peak excitation range of 500–600 nm and maximum emission at 600 nm. This optical configuration requires direct line-of-sight access to individual fibres within a thin illuminated plane. Therefore, the use of fabrics or garments obscures fibre-level motion and prevents simultaneous flow and fibre visualisation, which is a current limitation of the proposed method. Using this method, tracer particles illuminated by the laser were not visible, and only the fluorescent light from the yarns above 545 nm was recorded by the camera.

Yarn image series were pre-processed to reduce signal noise before analysis. Denoising included a contrast-limited adaptive histogram equalisation with a window size of 64 pixels. Then, a wavelet transform with a noise threshold between 0.55 and 0.61 was applied, thereby reducing background noise from the camera sensor in low-illumination conditions. The images were transformed to logarithmic scale to improve yarn and fibre visualisation, and a general histogram equalisation was applied. Finally, a 2-D adaptive noise-removal Wiener filtering with a window size of 9 pixels reduced image noise. After denoising, a 2D polynomial geometric transformation is applied to align fibre images with PIV images by matching points extracted from calibration planes taken before the recordings.

After pre-processing, fibre movement was tracked with optical flow, a widely used and extensively studied technique derived from computer vision, first introduced in 1951^49^. The method solves the brightness constancy constraint equation between two consecutive images, i.e., $\:\frac{\partial\:I}{\partial\:t}+u\frac{\partial\:I}{\partial\:x}\:+v\frac{\partial\:I}{\partial\:y}=0$, where 𝐼 represents pixel image intensity, 𝑢 and 𝑣 are the components of the optical flow vector in the horizontal and vertical directions, respectively, $\:\frac{\partial\:I}{\partial\:t}$ is the temporal derivative of 𝐼, and $\:\frac{\partial\:I}{\partial\:x}$ and $\:\frac{\partial\:I}{\partial\:y}$ are the spatial derivatives of 𝐼. Two well-known approaches can be applied to solve the equation. The first, known as the Lucas-Kanade^50^, assumes flow constancy across neighbouring pixels, solving the equation for a pixel patch by minimising the least-squares error between two consecutive image patches. On the other hand, Horn and Schunk’s^51^ method considers smooth flow variation between adjacent pixels, imposing a smoothness constraint on the entire image. In both scenarios, the results are velocity vectors for each pixel on the image, which can be used to track fibre movement and deformation and compare them with flow forcing from PIV results.

## Results and Discussion

One of the primary challenges in the current research was selecting a representative flow that accurately represents the dynamics of a typical washing machine. To the best of the authors’ knowledge, there is no descriptive study on the flow and turbulence characteristics of a washing machine on experimental or computational simulations, with only a reported use of PIV on a vertical-axis (top-loading) washing machine describing velocity profiles scarcely^52^. However, previous studies tracked garment dynamics inside horizontal-axis (front-loading) and vertical-axis washing machines. In the former type, a preceding research^53^ has shown that garment deformation is highly related to the Reynolds number (𝑅𝑒): the variation in the garment’s centre of mass position and stresses increase as 𝑅𝑒 increases. Regarding vertical-axis washing machines, experimental data tracking textiles^26^ have shown highly heterogeneous velocity profiles in the horizontal and vertical directions, but near homogeneity in the azimuthal (drum depth) direction. Furthermore, the garments are subjected to stagnation and high-velocity zones^26,54^. Following the mentioned literature, a VKSF, one of the canonical flows in fluid mechanics, was chosen because it presents a high-shear layer zone that encompasses large- and small-scale turbulent structures^55^ with instantaneous velocity variations^56,57^, which features velocity profiles in the textiles tracking studies. In this scenario, the fibre’s reaction to flow forcing under varying conditions can be investigated.

Another imperative flow characteristic is the Reynolds number, which relates inertial and viscous forces and can be used to compare flow characteristics between different experiments. To the best of the authors’ knowledge, Reynolds number in washing machines has not been adequately defined. Previous research has assumed 𝑅𝑒 to be equivalent to mixing vessels^53,58^ or Taylor-Couette flows^59^, whose geometries and dynamics are not analogous to a washing machine. For mixing vessels, it is accepted that $\:Re=ud/\nu\:=f{d}^{2}/\nu\:$. Notice that the characteristic length considers impeller diameter (𝑑) and omits 𝜋 from the characteristic velocity $\:u={\Omega\:}d/2=\pi\:fd$ based on experimental flow transitions from laminar to turbulent flow^60^. 𝜈 is water kinematic viscosity. However, for a vertical-axis washing machine, assuming 𝑑 and neglecting 𝜋 a priori cannot be justified, because there is no experimental data to validate flow transition. On the other hand, a Taylor-Couette geometry is characterised by a flow confined between two cylinders where at least one is rotating, generating an azimuthal flow^61^. In vertical-axis washing machines, the agitator (propeller) cannot be considered a cylinder, and the flow generated is not only azimuthal. Furthermore, the geometry of horizontal-axis washing machines differs significantly from that of either the Taylor-Couette or mixing vessel.

Here, it is proposed that for horizontal-axis washing machines, 𝑅𝑒_ℎ_ should be as outlined in Eq. (2), where $\:u={{\Omega\:}}_{max}{r}_{c}=2\pi\:{f}_{c}{r}_{c}$ is the characteristic velocity injecting energy into the flow, 𝛺_𝑚𝑎𝑥_ is the maximum angular velocity of the drum (rad/s), substituted in Eq. (2) by drum frequency 𝑓_𝑐_ (Hz), 𝑟_𝑐_ is drum radius, and 𝐿_𝑐_ is the characteristic length injecting energy to the flow, in this case paddle height, since flow separation is exerted mainly by paddles. Equation (3) describes 𝑅𝑒_𝑣_ for vertical-axis washing machines, where 𝑓_𝑐_ is agitator maximum frequency, and 𝑟_𝑐_ is agitator radius because the characteristic length and velocity are determined by agitator geometry (the drum is stationary), regardless of whether the gap between agitator radius and drum radius is minor than agitator radius.

2 $$\:R{e}_{h}=\frac{2\pi\:{f}_{c}{r}_{c}{L}_{c}}{\nu\:}$$

3 $$\:R{e}_{v}=\frac{2\pi\:{f}_{c}{r}_{c}^{2}}{\nu\:}$$

Given Eqs. (2) and (3), 𝑅𝑒 would differ depending on washing machine geometry and laundry cycle. For the horizontal-axis case, 𝑅𝑒 varies between 10,996 and 85,765 according to literature measurements of washing machines^54,58,62–68^, describing a turbulent flow. Concerning the vertical-axis scenario, available studies^53,59^ based their dimensions on experimental design rather than washing machines, leading to 𝑅𝑒 overestimations. A comprehensive review of magnitudes to calculate 𝑅𝑒 can be found in the Supplementary Information.

To test the method’s capabilities, three 𝑅𝑒 values within the range of horizontal-axis washing machines were experimented. In a VKSF $\:Re=\pi\:\left({f}_{1}+{f}_{2}\right){r}_{d}^{2}/\nu\:$, where 𝑓_𝑛_ is motor 1 and 2 frequencies, 𝑟_𝑑_ is disk radius, and 𝜈 is water kinematic viscosity at 20 °C. Therefore, 𝑅𝑒 is equal to 38,013, 50,684, and 63,355 for the three motor frequencies (see Table 2). The first motor frequency (0.5 Hz) is the minimum achievable velocity in the overhead stirrers. In contrast, the highest motor frequency (0.833 Hz) is limited by the camera sensors and the diaphragm’s opening, as the signal-to-noise ratio in the recorded images exceeds the threshold for denoising.

**Fig. 4:**
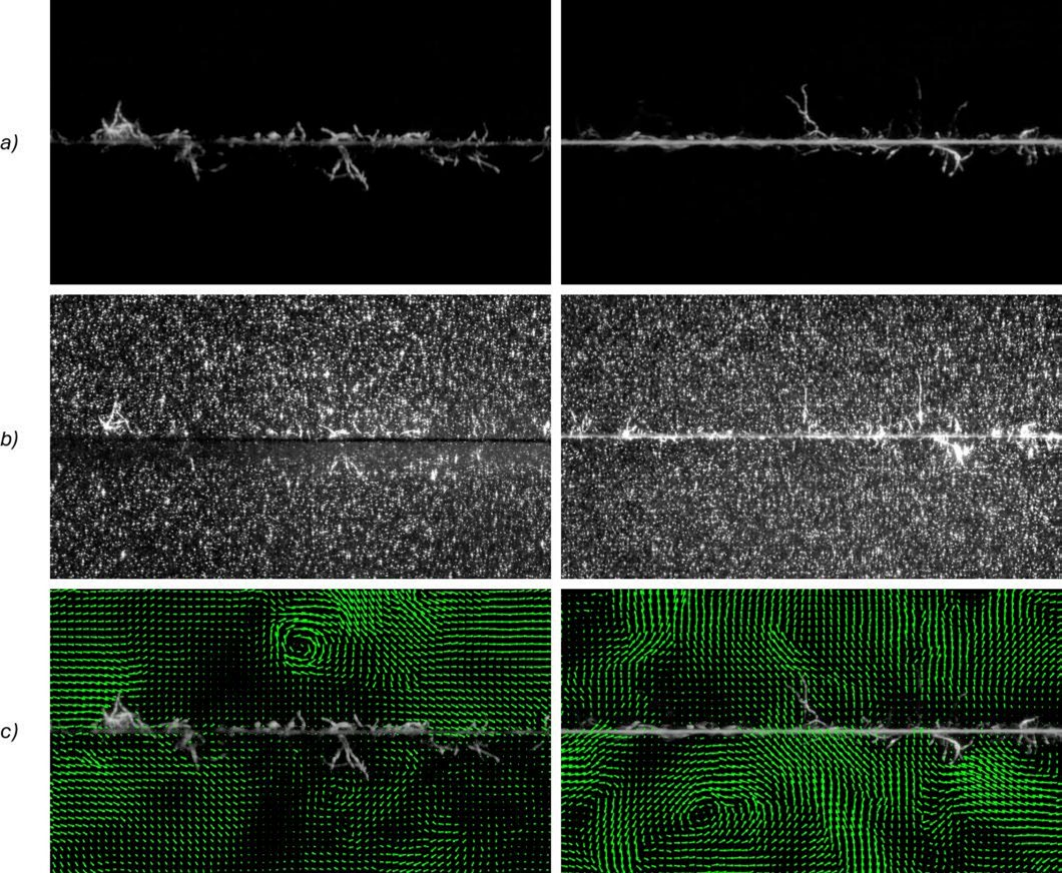
PET (left) and CO (right) experiments at 𝑅𝑒=38,013 for overhead stirrer motor at 𝑓𝑐 =0.5 Hz. (**a**) yarn images taken by a high-speed camera with a 545 nm optical filter to capture fluorescent light, making tracer particles seeded in the flow non-visible. (**b**) hollow-glass tracer particles used for PIV, taken simultaneously with a). (**c**) velocity vector reconstruction (green) showing the results from PIV analysis qualitatively overlayed on yarn images.

The proof-of-concept experiment results are shown in Fig. 4. The left column contains a PET yarn experiment, while the right column corresponds to a CO yarn sample. All images shown are 11.5% cropped from the original frame size to facilitate visualisation for readers, given the high resolution of the recorded images. Figure 4a) deploys yarn images after denoising process, where the hollow-glass particles seeded in the flow are non-visible due to a 545 nm optical filter in the camera; these images are used to track fibre movement. Figure 4b) shows hollow-glass particles using a second camera with no optical filter. These images are utilised for PIV analysis. The PIV images’ histogram is adjusted to highlight the foreground. Here, it is possible to observe the hollow-glass particles (small white points) along with the yarn and some fibres protruding from it. Fibres and yarn in Fig. 4a) and Fig. 4b) are aligned. The difference in yarn/fibre illumination is due to the cameras facing each other at 180 °, each capturing a yarn/fibre face. Figure 4c) depicts yarn images with overlayed velocity vectors (green lines). Longer lines represent higher velocity. Vortex formations are evident in both bottom images. Qualitative differences were observed between PET and CO fibres. Cotton fibres exhibited greater curvature and deformation under comparable flow conditions, consistent with their lower tensile stiffness and strength relative to PET, demonstrating the method’s applicability to materials with contrasting mechanical properties.

**Fig. 5:**
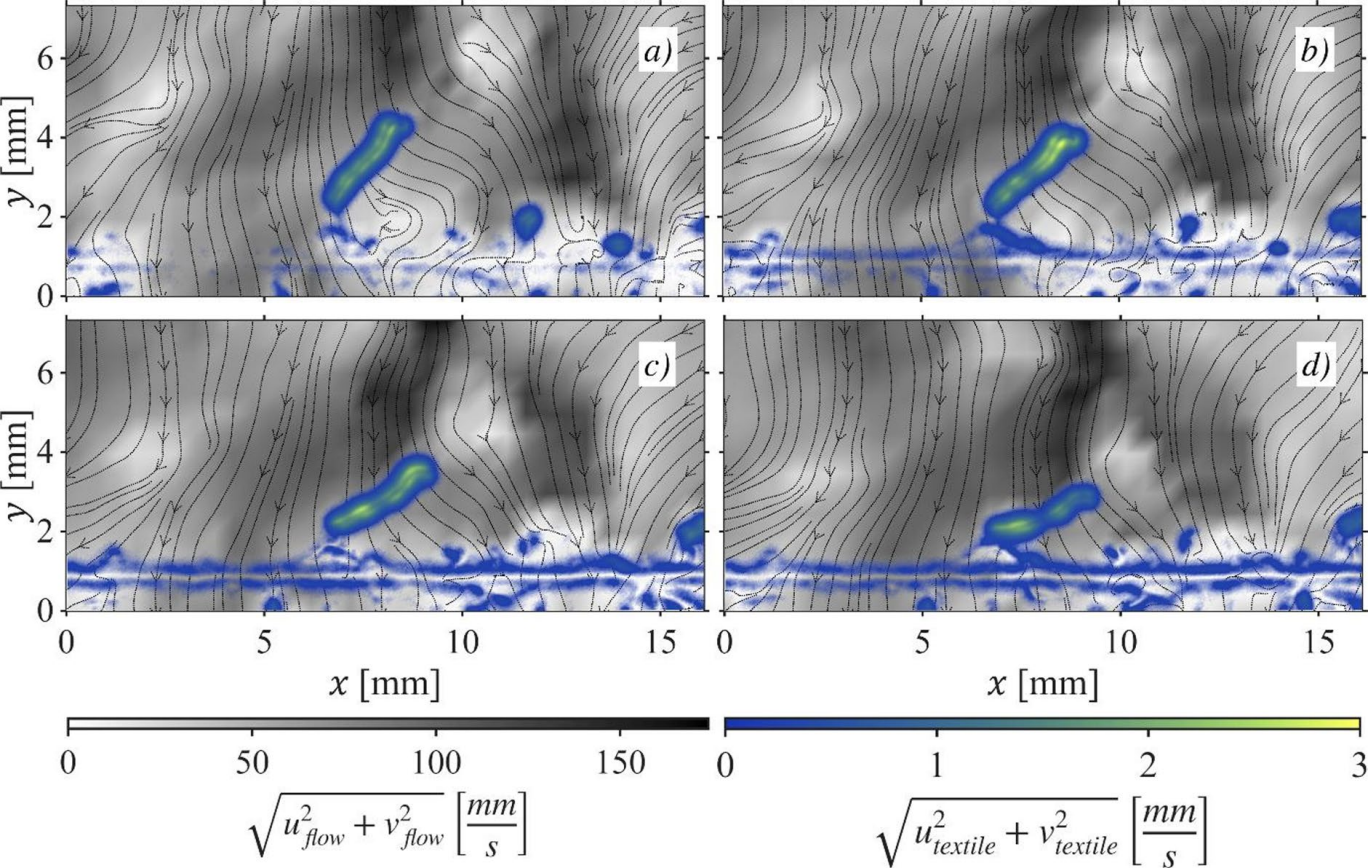
Flow and fibre (CO) timestep reconstructions at 𝑅𝑒=38,013 and overhead stirrer motor at 𝑓𝑐=0.5 Hz. Flow velocity is displayed by the colour scale from white to black. The black lines with arrows illustrate flow streamlines. The overlaid colourmap, ranging from blue to chartreuse-yellow, represents textile motion. Both colourmaps’ velocities are in mm/s. The indices a) to d) describe an attached fibre’s rotation and deformation. The timestep used to calculate velocities is 0.0025 s. Contrastingly, the timestep between shown images is 0.01 s.

Figure 5 displays a timestep reconstruction comparing flow and CO yarn motions. Flow velocity, obtained from PIV, is shown in the background gradient map, with grayscale colours, and flow streamlines in black indicating the flow direction. Optical flow was solved using Horn and Schunk’s^51^ method to obtain fibre velocity at each image pixel. The foreground palette represents the textile movement, from blue to chartreuse-yellow. The time interval between the shown images is 0.01 s. However, velocity vectors for flow and textile were calculated with a timestep of 0.0025 s using frames between each displayed reconstruction. The yellowish elongated figure is a fibre attached to the yarn forced by the flow. Between Fig. 5a) and Fig. 5b) the fibre rotates, forced by the flow, following its direction, with a pivot point close to the yarn at $\:x\approx\:6$, $\:y\approx\:1$ mm. In Fig. 5c) and Fig. 5d), the fibre continues its rotation, exhibiting deformation in its shape caused by the heterogeneous flow forcing acting along its length, as evidenced by the colour variations on the grayscale background.

Determining flow and fibre velocities simultaneously enables statistical analysis in both media, thus characterising the interaction between them as a coupled system. Fibre motion and deformation can be quantified for different materials and fibre lengths, including maximum curvature and rotation speed. Also, oscillatory movements have been observed in some fibres in several experiments, indicating the interaction with coherent turbulent structures. The rapidly changing dynamics, like the example shown in Fig. 5 with velocities of 3 mm/s for a fibre of 3.9 mm length, indicate high stresses in the fibre longitude but primarily in the pivot point close to the yarn, which can lead to fibre breakage by fatigue^69^ and eventual release. Additionally, the flow can be analysed to identify turbulence structures, such as vorticity and turbulent kinetic energy, as well as other parameters related to fibre properties, including length, diameter, and flexural rigidity.

The capacity to determine fibre oscillation and deformation introduces new mechanistic metrics. Oscillation amplitude, curvature, and frequency can be quantified directly from optical flow data, revealing how fibres respond to coherent turbulent structures. Larger oscillations and sharper curvatures correspond to higher flexural stresses, providing a measurable proxy for susceptibility to breakage.

Repeated oscillation cycles and deformation patterns can be directly linked to fatigue-driven breakage. Cyclic loading around fibre pivot points gradually accumulates damage, and, over time, this can exceed fatigue thresholds, leading to fragmentation. By measuring oscillation frequency and amplitude, the number of cycles to failure can be estimated using established fatigue models from material mechanics.

Further analysis could employ correlation and autocorrelation functions to characterise periodicity and coherence in fibre motion. Cross-correlation between flow fields (PIV) and fibre dynamics (optical flow) would clarify how turbulent structures force oscillations. Complementarily, autocorrelation would reveal whether fibre movements are random or display persistent periodicity. These tools expand the methodology’s capacity from qualitative demonstration to predictive modelling of fibre fatigue.

The physical and environmental implications of this methodology are significant. By quantifying fibre-flow interactions at this resolution, the method can enable the optimisation of washing-machine design and laundry cycles that can minimise turbulent stresses leading to minimal fibre detachment. Such adaptations could reduce fibre breakage and environmental release while extending textile lifetime, thereby delivering both consumer and environmental benefits.

Compared to existing parametric studies, the proposed methodology provides a deeper understanding of fibre breakage for natural (CO) and synthetic (PET) fibres (microplastics), utilising descriptive and inferential statistics to analyse fibre-flow interactions. Different laundry cycles can be simulated by adjusting the stirrer speed, ensuring controlled flow conditions. Furthermore, the yarn mounting system ensures repeatability by bringing the same tension for every yarn test. The frequency method used for wire tension was tested until the failure point was reached. The ultimate tensile strength presents an error of 4.5%, which is an acceptable margin for laboratory testing applications.

## Conclusion

This study directly addresses a central research gap. While many investigations have quantified total fibre release, the mechanisms of fragmentation and detachment remain poorly understood due to the lack of techniques capturing fibre-flow interactions under controlled conditions. By coupling PIV and optical flow, this methodology provides the missing experimental tool to observe, measure, and explain the mechanical pathways governing fibre release, including microplastics, at the level of individual fibre dynamics within turbulent flow fields. Rather than quantifying release, the approach resolves local fibre deformation and motion driven by hydrodynamic forcing, and cyclic deformation mechanisms that precede fragmentation and detachment, which are not covered by existing gravimetric methods and supporting upstream mechanistic understanding.

The results demonstrate that the proposed methodology enables fundamental observations of fibre–flow interactions and provides actionable insights. By identifying oscillation and deformation mechanisms that drive fibre breakage, it could be possible to design laundry cycles that reduce fibre release. This can be achieved either by modifying flow properties (e.g., reducing turbulence intensity, adjusting washing-machine rotation speeds) or by altering yarn structures (e.g., decreasing hairiness, optimising twist to suppress protrusion). Thus, the methodology bridges fluid-fibre physics with applied strategies to mitigate environmental microplastic pollution.

This approach idealises a complex phenomenon that includes textile-textile and textile-drum interactions, chemical additives, and air entrainment, all of which influence laundering. Additionally, experimental data from washing machines should confirm the transition from laminar to turbulent flow, validating the proposed Reynolds number definition. Nonetheless, controlled experimentation is essential to build a mechanistic baseline. By enabling non-invasive observation of fibre-flow interaction, this methodology provides a robust foundation for empirical studies in which variables such as detergents, water quality, or textile structures can be systematically explored to reduce fibre pollution, including microplastics.

## Supplementary Information

Below is the link to the electronic supplementary material.

Supplementary Material 1

## Data Availability

Data will be available upon request.

## Declarations

### Supplementary Information

1. Washing machine measurements available in literature to calculate Reynolds number, including paddle height, drum radius and frequency.
