# Supplementary material for: A novel methodology to study the release of fragmented fibres, including microplastics, in laboratory washing conditions
Source: Sci Rep. 2026 Mar 1;16:11493. doi: 10.1038/s41598-026-41563-7 (PMC13057226; doi:10.1038/s41598-026-41563-7)
Supplement: Supplementary file 1 — Supplementary Material 1 [file 41598_2026_41563_MOESM1_ESM.docx]

## Supplementary Information

1. Washing machine measurements available in literature to calculate Reynolds number, including paddle height, drum radius and frequency.

Table a: horizontal-axis (front-loading) washing machines spinning frequency (𝑓_𝑐_), drum radius (𝑟_𝑐_), and paddle height (𝐿_𝑐_) according to literature sources. Measurements were taken from washing machines unless it is mentioned otherwise.

| Source | 𝑓_𝑐_ min [Hz] | 𝑓_𝑐_ max [Hz] | 𝑟_𝑐_ min [cm] | 𝑟_𝑐_ max [cm] | 𝐿_𝑐_ [cm] |
| --- | --- | --- | --- | --- | --- |
| Ward, D. (2000). Modelling of a horizontal-axis domestic washing machine. *Journal of the Textile Institute*, *91*(2), 207-234. | 0.500 | 1.000 | 20.00 | 25.00 | NA |
| Park, J., & Wassgren, C. R. (2003). Modeling the dynamics of fabric in a rotating horizontal drum using the discrete element method. *Particulate Science and technology*, *21*(2), 157-175. | NA | 0.900 | NA | 23.75 | 3.5 |
| Tetik, T. U. Ğ. B. A., Yildiz, R. A., Labanieh, A. R., Yoruk, B., Kursun Bahadir, S., Kalaoglu, F., & Koncar, V. (2021). Hydrodynamic modeling of e-textile fabric washing behavior by the Coupled Eulerian–Lagrangian method. *Textile Research Journal*, *91*(9-10), 1117-1131. | 0.433 | 0.767 | NA | 25.91 | 4.5 |
| Yun, C., Choi, H. R., Park, S., & Park, C. H. (2019). The effect of fabric movement on washing performance in a front-loading washer V: Focusing on the role and shape of the lifter. *Textile Research Journal*, *89*(3), 364-374. | NA | 0.783 | NA | NA | 5.2 |
| Liu, G., Cui, Y., Wang, K., Wang, L., Wu, M., & Liu, H. (2025). The mechanism of microplastic fibers release in a front-loading washing machine. *Textile Research Journal*, 00405175241291216. | NA | 0.667 | NA | NA | NA |
| Zhao, X., Wang, C., Wang, L., Gong, H., & Ding, X. (2023). Performance of tapping washing in comparison with hand washing and front-loading machine washing for knitted wool sweaters. *Textile Research Journal*, *93*(3-4), 538-553. | 0.583 | NA | NA | 26.25 | NA |
| Zaman, S. U., Tao, X., Cochrane, C., & Koncar, V. (2021). E-textile systems reliability assessment—A miniaturized accelerometer used to investigate damage during their washing. *Sensors*, *21*(2), 605. | 0.250 | 0.642 | NA | 23.50 | NA |
| Liu, H., Gong, R. H., Xu, P., Ding, X., & Wu, X. (2019). The impact of rotational speed and water volume on textile translational motion in a front-loading washer. *Textile Research Journal*, *89*(16), 3401-3410. | 0.500 | 1.000 | NA | 24.50 | NA |
| van der Weg, P. B. (2019). Reduction of Environmental Impact of Drum Machine Washing. *Journal of Applied Mathematics and Physics*, *8*(1), 132-157. | 0.483 | 0.917 | 23.00 | 25.00 | NA |

Note:

- In some sources, more than one drum velocity was experimented, denoted in Table a as 𝑓_𝑐_ min (minimum) and max (maximum).
- In some sources, more than one drum radius was experimented, denoted in Table a as 𝑟_𝑐_ min (minimum) and max (maximum).
- NA stands for not available values.

Given Equation (2) to calculate 𝑅𝑒 number for horizontal-axis washing machines (repeated below):

$$Re_{h}=\frac{2\pi f_{c}r_{c}L_{c}}{\nu}$$

Where:

$f_{c}$ [Hz]: drum frequency.

𝑟_𝑐_ [cm]: drum radius.

𝐿_𝑐_ [cm]: paddle height.

Minimum and maximum values from Table a are used to calculate Reynolds number for horizontal-axis (front-loading) washing machines (considering water at 20 °C):

$$Re_{v}=10,995.57-85,765.48$$

Table b: vertical-axis (top-loading) washing machines spinning frequency (𝑓_𝑐_), and agitator radius (𝑟_𝑐_) from experimental-based sources. The measurements are not obtained from washing machines.

| Source | 𝑓_𝑐_ min [Hz] | 𝑓_𝑐_ max [Hz] | 𝑟_𝑐_ [cm] |
| --- | --- | --- | --- |
| Akcabay, D. T., Dowling, D. R., & Schultz, W. W. (2014). Clothes washing simulations. *Computers & Fluids*, *100*, 79-94. | NA | 2.000 | 18.6 |
| Loyola, F. R., e Silva Jr, W. D. L., Campos, L. G., & Hermes, C. J. (2018). Rheometric assessment and numerical simulation of steady-state and periodic flows of fabric-water mixtures in household top-load washing machines. *Chemical Engineering Research and Design*, *137*, 273-290. | 0.083 | 2.000 | 11.0 |

Considering Equation (3) to calculate 𝑅𝑒 number for vertical-axis washing machines (repeated below):

$$Re_{h}=\frac{2\pi f_{c}r_{c}^{2}}{\nu}$$

Where:

$f_{c}$ [Hz]: agitator frequency.

𝑟_𝑐_ [cm]: agitator radius.

Minimum and maximum Reynolds numbers for vertical-axis (top-loading) washing machines considering Table b measurements, with water at 20 °C, are displayed below:

$$Re_{v}=6,310.20-434,746.16$$

**Measurements from available literature for vertical-axis (top-loading) are not based on washing machines. Instead, dimensions and frequencies are experimentally designed. Therefore, 𝑅𝑒 estimation should be considered with caution.**
